# Case Report: Suspected “stiff dog syndrome” in a Maltese dog

**DOI:** 10.3389/fvets.2025.1613131

**Published:** 2025-06-13

**Authors:** Valentina Buffagni, Francesca Fidanzio, Diletta Dell’Apa, Fabio Tummolo, Marco Bernardini, Ezio Bianchi

**Affiliations:** ^1^Department of Veterinary Medical Sciences, University of Parma, Parma, Italy; ^2^Anicura Tibaldi Veterinary Clinic, Milano, Italy; ^3^Euroimmun Italia, Padova, Italy; ^4^Anicura I Portoni Rossi Veterinary Hospital, Zola Predosa, Bologna, Italy; ^5^Department of Animal Medicine, Productions and Health, University of Padua, Legnaro, Italy

**Keywords:** neuronal excitability, muscle hypertonicity, stiff dog syndrome, IVIg, CBD

## Abstract

A Maltese dog was presented with a stiff gait, secondary to muscle hypertonicity, affecting the axial and proximal appendicular muscles, which had progressively worsened over the last 4 years, associated with episodes of muscle spasms. Neuroanatomical localization was upper motor neuron (UMN) or generalized neuromuscular system. Cerebrospinal fluid (CSF) analysis was normal. Magnetic resonance imaging (MRI) of the brain and cervical spinal cord was performed and showed hypoplasia of the dorsal part of the left hippocampus, unchanged compared to the MRI performed 4 years earlier, and mild C6–C7 disk extrusion, with no evidence of compression of the spinal cord. Conscious electromyography showed continuous motor unit action potentials (MUAPs) in agonist and antagonist muscles. Indirect immunofluorescence (IFT) detected the presence of antibodies against glutamic acid decarboxylase (GAD). These findings were consistent with a human condition called “Stiff Person Syndrome” (SPS). A condition similar to SPS has only been described once before in a Beagle dog (“Stiff Dog Syndrome”). A therapeutic protocol based on human guidelines for SPS was initiated with a partial improvement. “Stiff Dog Syndrome” (SDS) is a possible cause of muscle hypertonicity and spasms in dogs.

## Introduction

Stiff person syndrome (SPS) is a rare immune-mediated disorder usually diagnosed in adulthood (1–3). The classical form, which accounts for 70% of cases, is characterized by an insidious onset and progression of muscular rigidity in the trunk, which spreads to the limbs and often leads to orthopedic diseases, such as non-traumatic bone fractures and joint luxation (3–5). Other phenotypes have been described, such as partial SPS, characterized by stiffness in only one limb (stiff limb syndrome), and SPS plus, in which brainstem or cerebellar signs are added to the clinical signs of the classic form (1).

The clinical signs are mainly due to reduced activity of the enzyme glutamic acid decarboxylase (GAD), which is responsible for the synthesis of *γ*-aminobutyric acid (GABA), the main inhibitory neurotransmitter of the nervous system (2, 3). The pathogenesis is not entirely understood. However, the presence of antibodies against GAD is thought to cause a reduction in GABA production, a decrease in GABAergic transmission, and consequent hyperactivity of motor neurons, resulting in simultaneous contraction of agonist and antagonist muscles (2, 6).

There is no single specific neurological sign or laboratory test for the diagnosis of SPS (1). The diagnostic criteria for SPS include clinical presentation, neurological examination, exclusion of other neurological disorders, electromyographic evidence of simultaneous motor unit action potentials (MUAPs) in agonist and antagonist muscles, and positive serology for anti-GAD antibodies (1–3).

A condition similar to SPS has been described in horses (“Stiff Horse Syndrome,” SHS) (7, 8) and in a Beagle dog (“Stiff Dog Syndrome,” SDS) (9). This case report describes a case of a Maltese dog diagnosed with suspected SDS based on clinical, electrodiagnostic, and laboratory findings, alongside the exclusion of other causes of muscle stiffness. The report also includes the dog’s response to SPS-based treatments and long-term follow-up (2 years and 5 months).

## Case presentation

An 8-year-old male, 4.8 kg, Maltese dog was presented with a 4-year history of slowly progressive stiff gait and superimposed episodes of spasm of the axial muscles, especially in the cervical region, usually lasting 2 to 3 days. No autonomic or post-ictal signs were reported by the owner during these episodes. In previous years, the patient had undergone orthopedic consultations, radiographs of the limbs, and ultrasonography of the shoulder muscles, which revealed the presence of bicipital tenosynovitis of both forelimbs and patellar luxation. Four years prior to referral to our institution, a neurological examination and MRI of the brain were performed to investigate the causes of the reported clinical signs. Various treatments were tried, including steroids and non-steroidal anti-inflammatory drugs, but there was no improvement. At the time of referral to our institution, the general physical examination was within normal limits. Neurological examination revealed severe muscle hypertonicity affecting axial and both flexor and extensor muscles in limbs, resulting in stiff gait, exercise intolerance, and muscle hypertrophy. Mild lameness (Grade 2) was present in the forelimbs. Muscle hypertonicity was exacerbated by stress and manipulation. Mental status, behavior, postural reaction, cranial nerves, and spinal reflexes were normal.

The neuroanatomical localization was the upper motor neuron (UMN) or generalized neuromuscular system. Differential diagnoses included inflammatory, non-infectious, immune-mediated (stiff dog syndrome, SDS), metabolic (“Cushing’s myotonia” and hypocalcemic tetany), and degenerative neuromuscular (congenital myotonia, neuromyotonia, late-onset muscular dystrophy) disorders. Although less likely, inflammatory infectious, degenerative, or neoplastic disorders affecting the central nervous system (CNS) and a movement disorder such as paroxysmal dyskinesia associated with generalized dystonia could not be completely excluded. Because of the chronicity of the clinical signs, tetanus was considered very unlikely (10).

Hematology, biochemistry, and urinalysis were unremarkable. Serologies for *Neospora caninum* and *Toxoplasma gondii* were negative. Abdominal ultrasound and chest radiographs, performed mainly to investigate the presence of neoplastic disease, were also unremarkable. A low-dose dexamethasone (0.01 mg/kg, IV) suppression test was performed. The results were not consistent with hyperadrenocorticism: basal cortisol (before dexamethasone) was 2.93 μg/dL (range 1–5 μg/dL) and less than 1.4 μg/dL at 4 and 8 h after dexamethasone injection.

A high-field MRI (1.5 Tesla Vantage Elan, Canon Medical Systems Europe B.V., Netherlands) of the brain and cervical spinal cord was then performed. Brain MRI showed hypoplasia of the dorsal part of the left hippocampus and dilatation of the left lateral ventricle. These findings were similar to MRI performed 4 years earlier. A cervical spine MRI showed mild C6–C7 intervertebral disk extrusion associated with ventral spondylosis and no evidence of compression of the spinal cord. Cerebellomedullary cerebrospinal fluid (CSF) analysis was within normal limits (white blood cells 0, range 0–5/mm^3^; protein 30 mg/dL, range 0–30 mg/dL).

**Figure 1:**
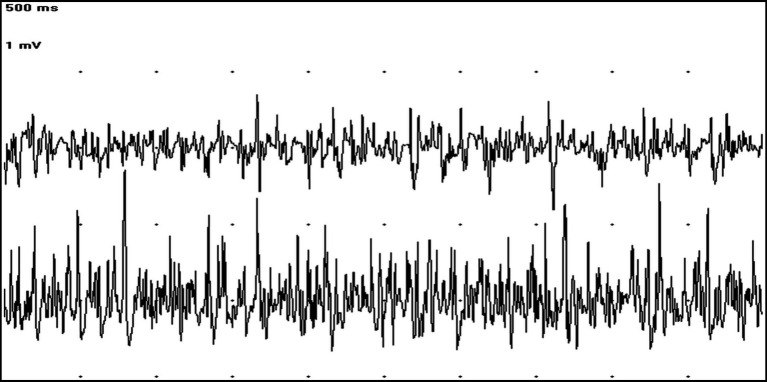
EMG tracings showing at the same time MUAPs in the cranial tibialis (trace above) and gastrocnemius muscles (trace below) (50 ms/Div; 1 mV/Div).

An electrodiagnostic study including electromyography (EMG) of axial and appendicular muscles, motor and sensory nerve conduction studies, F waves, repetitive nerve stimulation, and cord dorsum potential was carried out to determine the nature and origin of the muscle hypertonicity. Spontaneous electrical activity recorded by conscious EMG showed continuous MUAPs in both agonist and antagonist muscles (Figure 1). This spontaneous pathological activity ceased during general anesthesia. The results of the other electrodiagnostic tests were within normal limits.

**Figure 2:**
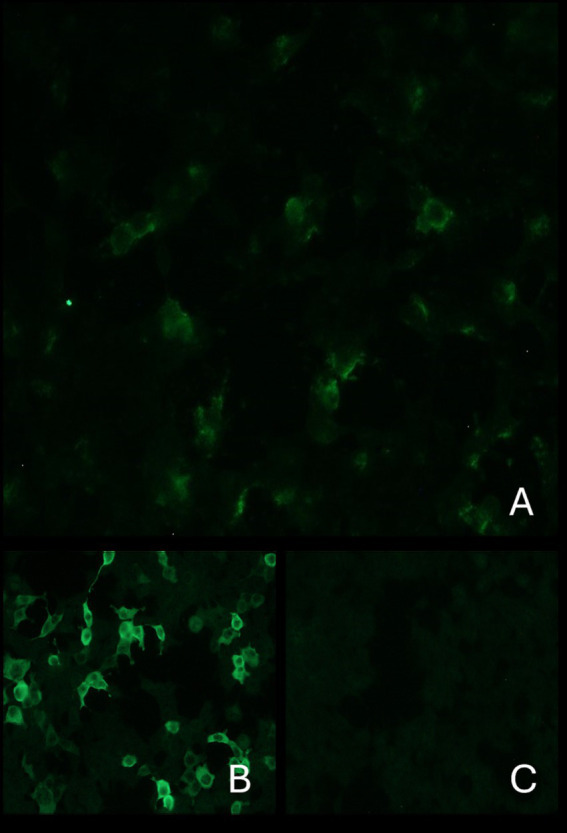
Indirect immunofluorescence: The top image shows the presence of anti-GAD antibodies in the serum of the current case **(A)**. Images below: on the left positive control **(B)** and on the right negative control **(C)**. Antibodies directed against GAD react with transfected cells of the substrate. These cause a fluorescence pattern of the cytoplasm. The cell nuclei are only faintly stained.

A semiquantitative indirect immunofluorescence technique (IFT) based on transfected cells (Euroimmun Ag, Germany, Code N. FA1022-1005-50) was performed, according to the manufacturer’s instructions, to evaluate the presence of anti-GAD antibodies in serum and CSF. The serum was diluted (1:10), and CSF was used undiluted. Negative controls consisted of serum obtained from a normal dog, in addition to the human one, provided by the manufacturer. This test confirmed the presence of anti-GAD antibodies in the serum (Figure 2), but not in the CSF. Based on clinical and diagnostic findings, stiff dog syndrome was suspected (9).

According to human guidelines for SPS (1–4), treatment with clonazepam 0.5 mg/kg/12 h (Rivotril, Cheplapharm Arzneimittel GmbH) was started but discontinued after 2 days due to excessive sedation and incoordination. Gabapentin (Gabapentin, Teva Pharma S.r.l) was then prescribed at 10 mg/kg/8 h. This was also discontinued after a few days due to excessive sedation. A galenic formulation of 5% cannabidiol (CBD) was then administered at 2 mg/kg/12 h, favoring slight generalized muscle relaxation.

With this therapeutic regimen, the clinical signs did not progress until 1 year later, when the dog returned for neurological consultation for an episode of right-sided torticollis. The neurological examination revealed thoracolumbar kyphosis, cervical pain, and spasms of the axial cervical muscles associated with the previously observed generalized muscle hypertonicity and hypertrophy. Mentation, postural reaction, spinal reflexes, and cranial nerve examination were normal. Hematology and biochemistry were unremarkable. The owner declined a third cervical MRI. Therapy with meloxicam (Meloxoral, Dechra Veterinary Products S.r.l., 0.1 mg/kg/24 h for 7 days) was started with resolution of the torticollis after 3 days, but persistence of generalized muscle hypertonicity. Suspecting that the clinical signs observed were secondary to a worsening of SDS, and in an attempt to better control the neurological signs and prevent episodes of muscle spasms, a constant-rate infusion of intravenous immunoglobulin (IVIg) (Privigen, CSL Behring S.p.A.) was administered at a dose of 1 g/kg over 8 h, as reported in human literature for SPS (4). A slight improvement in clinical signs was observed during the first few weeks after the infusion.

One month after the immunoglobulin treatment, neck muscle spasms and torticollis relapsed. These clinical signs improved with symptomatic treatment with a 7-day course of paracetamol 10 mg/kg/12 h (Tachipirina, Angelini Pharma) and gabapentin 8 mg/kg/8 h for 30 days.

The long-term therapy consisted of gabapentin 10 mg/kg/12 h and 5% CBD oil 2 mg/kg/12 h. This was further increased up to 4 mg/kg/12 h due to deterioration of the clinical signs. At the time of writing, 2 years and 5 months after the initial assessment, the progression of the disorder has resulted in further impairment and stiffness of the dog’s gait, worsening of generalized muscle hypertonicity and hypertrophy (Supplementary Video 1) and a slow increase in the frequency of episodes of cervical muscle spasms and torticollis (Supplementary Video 2), which appear to be particularly associated with emotional stress and cold weather. Cervical muscle spasms occur 5–6 times a year, especially in winter, and are controlled by increasing the dose of CBD up to 7 mg/kg/12 h for a few days. The owner has reported better control of clinical signs and a reduction in spasm duration with this therapy and considers the dog’s overall quality of life to be good.

## Discussion

Muscle stiffness can be associated with CNS, peripheral nervous system (PNS), and muscle abnormalities (11). Electrodiagnostic studies can help characterize and identify the cause of muscle hypertonicity (11, 12). In particular, muscle stiffness caused by PNS or muscle disorders is usually associated with spontaneous activity at EMG that persists during general anesthesia and may have specific characteristics (e.g., myotonic discharges and neuromyotonic discharges) (13, 14). On the contrary, spontaneous discharges on conscious EMG that disappear under general anesthesia, as in the dog described in this case report, suggest a UMN origin (12). Given these findings and normal blood muscle enzymes, a muscle biopsy was not included in the diagnostic work-up.

In this dog, the continuous MUAPs recorded on conscious EMG in both agonist and antagonist muscles, together with the history, clinical presentation, neurological examination, and the presence of anti-GAD antibodies, were suggestive of SDS, the canine equivalent of SPS (1, 9). Indeed, the diagnostic criteria for SPS are the presence of stiffness in limb and axial muscles evoked by tactile and auditory stimuli, EMG evidence of continuous motor unit activity in agonist and antagonist muscles, positivity for anti-GAD antibodies in serum and/or CSF, and the exclusion of other neurological and non-neurological diseases that can cause muscle stiffness (1, 3). To investigate other possible CNS causes of muscle stiffness, an MRI of the brain and cervical spinal cord was performed and compared with an MRI of the brain performed 4 years earlier. The hypoplasia of the dorsal part of the left hippocampus was present with similar characteristics and extent to the previous MRI. A non-evolutive, possibly malformation disorder was suggested by the stability of the brain abnormalities (15). Furthermore, since the hippocampus does not play a primary role in determining muscle tone, we considered a correlation between the abnormalities detected on MRI and progressive neurological signs of the dog to be unlikely (16).

The location and apparent lack of compression caused by the C6–C7 disk extrusion also did not explain the findings on neurological examination of the dog at initial evaluation. A limitation of this case report is the lack of follow-up MRI of the cervical spine when, 1 year after the initial evaluation, there was a worsening of episodes of spasm of the axial muscles, particularly cervical pain and torticollis. Despite the normal neurological examination and the absence of signs of inflammation in the hematology and biochemistry, we cannot exclude that the progression of clinical signs at that time was the result of conditions such as compressive myelopathy or discospondylitis. Compared to the Maltese subject of this case report, the Beagle of the previous report suspected of SDS had a more acute onset of neurological signs and a better response to therapies and outcomes (9).

In that case, spontaneous or stimulus-induced episodes of generalized muscle spasms, lasting 1–10 min, started 3 weeks before and resolved 25 days after the first neurological consultation. The episodes were superimposed on persistent rigidity of the axial and proximal appendicular muscles, resulting in lordosis. Only a mild stiff gait remained 100 days after the initial evaluation. Neurological examination was normal at 18 months (9). Our dog had a longer history (at least 4 years) of slowly progressive stiff gait and superimposed episodes of spasms of the axial muscles. The spasms, which usually lasted 2–3 days, mainly affected the cervical region, seemed to be associated with manipulation, stress, and cold, and were progressively more frequent. Two years and 5 months after the initial evaluation, neurological signs are still present and only partially controlled by therapies.

The classic phenotype of SPS includes stiffness and spasms predominantly of the axial and proximal appendicular muscles (3). The signs are often asymmetrical and slowly progressive and include lordosis, cervical stiffness, gait problems, and pain. Patients with SPS often have concomitant orthopedic disorders and severely reduced quality of life (3–5).

As reported in SPS (3–5), in addition to muscle hypertonicity, orthopedic diseases complicated the gait difficulties in our case. In particular, bilateral bicipital tendinopathy, a condition that usually affects middle-aged to older dogs of medium to large breeds (17), was first diagnosed when the dog was 6 years old.

Anti-GAD antibodies are present in the serum and/or CSF of 60–80% of patients with SPS (3). In particular, high titers of anti-GAD65 antibodies (> 10,000 IU/mL) are a specific marker for SPS but may also be associated with other neurological disorders such as cerebellar ataxia, epilepsy, and limbic encephalitis (1, 18). In addition to SPS, lower levels of anti-GAD65 have been reported in other autoimmune diseases such as type 1 diabetes mellitus, which is present in approximately 35% of SPS patients, or in tumors (3, 19). In the case of the suspected SDS previously described, anti-GAD65 was detected in serum and CSF (9). As in this case report and in the horse with suspected SHS (7–9), in the absence of a validated test for these species, we used a test in human medicine for the diagnosis of SPS.

The IFT kit used in our case provides a semiquantitative measure of anti-GAD65 antibodies. For human serum, this assay, based on transfected cells expressing GAD65, is positive in 100% of samples with anti-GAD65 antibody concentrations greater than 10,000 IU/mL and negative in 96% of samples with concentrations less than 10,000 IU/mL (18). Therefore, we suspected that our dog had a high serum titer of anti-GAD65 antibodies. Other autoimmune or neoplastic diseases were excluded based on clinical signs, blood tests, abdominal ultrasound, chest X-ray, and long-term follow-up.

In human medicine, there is a stepwise therapeutic approach to SPS based on the pathophysiology of the disease (4). First-line therapy includes GABAergic drugs such as benzodiazepines (e.g., diazepam and clonazepam), which have muscle relaxant and sedative/anxiolytic effects, and antiepileptic drugs (e.g., gabapentin and levetiracetam) (4). Medications to treat spasticity, such as baclofen, are often given in combination with benzodiazepines (4). Immunomodulatory/immunosuppressive therapies are used as second-line treatments for severe or refractory SPS. Intravenous immunoglobulin is often the first treatment in this drug group (3). Plasmapheresis and rituximab are other options (2–4). Immunosuppressive drugs, such as corticosteroids, have been disappointing in the treatment of SPS (2, 4). Furthermore, in the dog in this case report, corticosteroids have been of limited benefit. Other immunosuppressants (azathioprine, methotrexate, cyclophosphamide, or mycophenolate mofetil), routinely used as maintenance therapy in other autoimmune neuromuscular diseases, have also been unsatisfactory in SPS (2, 4).

Despite difficulties in objectively assessing improvement in stiffness and spasms, the majority of patients with the classic form of SPS respond to treatment (3). Nevertheless, full recovery is usually not achieved, and relapses or slow progression of clinical signs over time are often reported despite treatment (1, 3, 4).

In the dog in this case report, benzodiazepines had to be discontinued after a few days because of adverse effects. Gabapentin, initially discontinued due to its sedative effect, was gradually reintroduced to treat spasms and then as a chronic therapy. Due to the dog’s apparent high sensitivity to the sedative effects of centrally acting drugs, baclofen and levetiracetam have never been tried. Other antispasmodics, such as methocarbamol or dantrolene, were not administered because they are less commonly used in SPS (3, 4) or were not found effective in SDS and stiff horse syndrome (8, 9).

The dog received a single infusion of IVIg with mild clinical improvement. It is possible that better control of muscle stiffness and spasms could have been achieved with an infusion of 2 g/kg administered over 2–5 days, as suggested in SPS (2–4). The owner declined this protocol for economic reasons. An improvement in muscle stiffness was associated with chronic oral administration of CBD oil. The use of cannabis derivatives has been reported to have a beneficial effect on muscle stiffness in some patients with severe forms of SPS (20, 21). The improvement may be related to the action of CBD as an allosteric modulator of GABA receptors (increasing the affinity of GABA for its receptor), which has been demonstrated in preclinical studies (22).

By adding a 1 g/kg IVIg infusion to therapy with centrally acting muscle relaxants and antiepileptics, a rapid and long-lasting improvement of clinical signs was achieved in the Beagle with suspected SDS (9). In this dog, all therapies were discontinued after a few months and the dog was neurologically normal 18 months after the initial evaluation.

The better outcome and response to therapy in this dog compared to the Maltese subject of our case report may be due to the shorter interval between the onset of clinical signs and immunotherapy (9). In our case, the presumptive diagnosis of SDS was made approximately 4 years after the onset of the first clinical signs. Consequently, treatments, including IVIg, were started when rigidity and muscle spasms were already very severe. Some authors suggest a better outcome when intravenous immunoglobulins are used early in the course of the disease in patients with SPS (1). In addition, the prolonged muscle spasms may have caused ischemic changes in the muscle tissue, which may have contributed to the severity of the clinical signs and the lack of response to treatment (23). Since an MRI was not performed immediately prior to the IVIg infusion, we cannot exclude the possibility that the reduced response to this therapy was due to the presence of concomitant cervical spine pathology.

In conclusion, SDS should be included in the differential diagnoses of dogs presenting with progressive generalized hypertonicity of the axial and proximal appendicular muscles with superimposed episodic muscle spasms. Once the other possible causes of muscle stiffness have been ruled out, an EMG should be performed both on the awake patient and under general anesthesia to confirm the origin of the stiffness in the UMN. The presence of anti-GAD antibodies in serum and/or CSF may support the diagnosis of SDS in these cases. Further studies are needed to determine the true incidence, spectrum of clinical features, and ideal treatment protocol for this disorder in dogs.

## Data Availability

The raw data supporting the conclusions of this article will be made available by the authors, without undue reservation.

## Supplementary material

SUPPLEMENTARY VIDEO 1 Worsening of gait stiffness and generalized muscle hypertonicity and hypertrophy over the years.

SUPPLEMENTARY VIDEO 2 The dog during two different episodes of cervical muscle spasms and torticollis.
